# Dr. Paris and Mr. Fonblanque on Medical Jurisprudence

**Published:** 1823-09-01

**Authors:** 


### VIII.

*Medical Jurisprudence.* By J. A. Paris, M.D. F.R.S. F.L.S. Fellow of the Royal College of Physicians; and J. S. Fonblanque, Esq. Barrister at Law. In three Volumes, 8vo. London, 1823.

Law and Physic have here joined hands, for the first time, we believe, in this country. Well! far be it from our wish to break either law or love matches; but still we adhere to our former opinion that there is no *necessary* connexion between the medical evidence delivered in a court of justice and the legal award that awaits the case. We will just take one or two examples from among those events which most frequently bring the medical practitioner in contact with the gentlemen of the long robe—viz. infanticide and poison. Will all the legal knowledge in Coke upon Littleton assist the physiologist in ascertaining the marks of life or causes of death in the one case;—or in detecting the poisonous substance with its train of consequent lesions in the other? In various countries there are various laws and various punishments for the same crime; yet the bearings of the physiological or pathological question are not affected thereby. We conceive that the justiciary consequences of crime or guilt, be they trifling or terrible, should have no operative influence on the medical investigation. If it were asked by the judge —"Sir, are you aware of the vast importance which attaches to your evidence in the case before us?" This would be the answer—"Sir, I am aware only of the importance of *truth*, and to that I shall adhere, divesting my mind of all consequences that may result from that truth being made evident."

We find that Sir William Blackstone entertained opinions similar to our own. "For the gentlemen of the faculty of physic," says he, "I must frankly own that I see no special reason why they in particular should apply themselves to the study of the law; unless in common with other gentlemen, and to complete the character of general and extensive knowledge—a character which their profession beyond others has remarkably deserved." It is true, Sir William afterwards hints that it would be no bad thing if physicians made themselves "*acquainted with the doctrine of last wills and testaments;*" thereby insinuating, we suppose, that the doctor should make the patient's will before he writes the prescription—a precaution which we admit to be very proper on many occasions. We must say, however, that if some doctors were obliged to draw up the last testaments of all the patients for whom they sign passports to the shades below, they would have work enough on hand.*

The authors of the work before us have given some curious reasons for the study of the law in connexion with medicine; among other this—that an accoucheur "unacquainted with the law of tenant by the courtesy" would be unable to satisfy his own conscience, ten years subsequent to an accouchment, if called upon to say whether the child was born alive or dead—and why?—because if unacquainted with the above law he—"would scarcely note whether a child, certainly dead within a minute of its birth, did in that period, move a limb or open an eye." This is nice navigation! Another reason for this junction of law and physic will seem rather unsatisfactory to all those who reside beyond the sound of Bow Bells.

"Can the President and Censors of the College of Physicians execute their power of fine and imprisonment; can they restrain unlicensed intruders, or *punish the bad practices of ignorant pretenders*, without some study of the law?" *Introduction.* xlvii.

That the College of Physicians will watch over their rights and privileges without any other stimulus than their charter, we have not the smallest doubt; but that the whole of Black-

---

* The above sentiments coincide with those of one of the best writers on Medical jurisprudence which this country has produced—Dr. Gordon Smith.

"In the courts of Great Britain, the physician appears for the most part in the simple capacity of a witness. He is generally examined *viva voce*, either as to his knowledge of a particular event, or his opinion on a fact that may be submitted to him; and to this exposure every member of the profession is equally liable. He is required to prepare himself by no course of study foreign to that of his proper profession, and to observe no formalities, but those of prudence and decorum. Juridical disputation and legal casuistry can hardly combine with medical reasoning, or illustrate the laws of our physical economy. It is the *prudentia Medicinæ* rather than the *prudentia Juris* that we are bound to cultivate—even with a view to Forensic application." 3.

stone's Commentaries would induce, or indeed enable them to "*punish the bad practices of ignorant pretenders*," no man in his senses could for a moment suppose.

Our authors, in fact, are able to adduce but a few vague, general, and unconvincing arguments in favour of this union of legal and medical knowledge in the physician or surgeon, and therefore we shall make but one more remark upon the subject. We have not the smallest objection that those, whose genius and talents are sufficiently comprehensive, should embrace the whole circle of the sciences—with this short proviso, that they first make themselves master of their own profession. Now, as the phenomena of disease and the operation of remedies alone, would require a whole life time to be well familiarized to the practitioner, we submit it to his prudence whether he should squander his time upon *legal* studies, that he may never have any occasion for, when such various and important branches of his own profession require his unremitting cultivation.

But while we object to the study of the law as a *necessary* branch of medical education, we urge the propriety of studying what is called forensic medicine, with more assiduity than has yet been manifested in this country. Not that we can coincide, in the following too sanguine statement of our authors.

"So rapid, however, has been the progress of the leading branches of medical knowledge during the last ten years; and so successfully have they disentangled themselves from the many fatal fallacies with which they were encompassed, that the general prejudice against their practical utility, in advancing the administration of justice, must gradually subside, and the study of forensic medicine become universally popular." *Introduction.* xxviii.

We should consider physiology, pathology, and therapeutics as among "the leading branches of medical knowledge;" but we confess we have looked around us in vain for those gigantic strides in these or indeed any other branches of medical science, which are said to have been made during the last ten years. Even in chemistry, the most progressive of all the collateral branches of medicine, we have not heard of any very recent and brilliant discoveries that are likely to "advance the administration of justice," unless it is that of Mr. Farrady, who, say our authors, "by a happy generalization has annulled the supposed characteristic distinction between *gas* and *vapour*."*

With the view of arousing our brethren to increased ener-

* Introduction, p. 42.

gy in the study of legal medicine, we propose to carry the subject through several of our succeeding numbers, by which means we shall be enabled to lay before our readers so very comprehensive an analysis of the interesting work under review, as shall form in itself no bad epitome of the subject, and thus secure a wider and more general perusal of the various topics discussed than has perhaps ever been effected before in this country and its scattered dependencies. We do not despair of presenting a connected and tolerably clear view of these volumes, because we perceive that, whether from the contagion (we may safely use this term as there is actual *contact*) of legal circumlocution, or some other cause, the language, though always easy and sometimes elegant, is far from that terse and idiomatic style which best suits an elementary and didactic work in any science. For the reasons already stated we shall be very sparing of the purely legal portions of the volumes before us, and indeed of some other portions, to which we imagine our authors have attached unnecessary importance, as will be shewn anon.

The work is divided into three parts—the *first* comprehending the enumeration of the different medical corporations, with an account of their charters, powers, and privileges, together with the subjects of medical police. The *second* comprehends all those subjects connected with medical evidence, as applicable to civil and ecclesiastical suits. The *third* discusses the enquiries which are necessary to medical evidence, as applicable to criminal cases.

Of the 71 pages dedicated to the three medical corporations of physicians, surgeons and apothecaries, 53 pages are appropriated to the College of Physicians,* 5 to the College Surgeons, and 13 to the Apothecaries.

We are of opinion, and the public will easily decide, that our authors have shewn rather too much anxiety to bring forward all the old records, charters, and laws of the College of Physicians. They have much overdone the thing, both in matter and manner, the latter of which is, in many instances, such as to generate more gall than good humour among the members of the profession practising as physicians in these realms by virtue of different diplomas. We are extremely sorry to see this, because we profess ourselves friendly to the constituted authorities and regular institutions, regarding them as the best bulwarks against empiricism, and the surest means of raising the science in the eyes of the world. But we wish

* In the Appendix, No. I. there are about 150 pages more dedicated to college affairs.

also to see the College of Physicians (and we acknowledge with pleasure that the great majority of its fellows do so) enjoy their rights, privileges, and prerogatives, with that sense of dignity and urbanity which comports with a liberal and enlightened profession in the nineteenth century. But to our subject.

It was our Eighth Henry who first granted a charter to the College of Physicians, in the year 1523. We shall not stop to pourtray the different clauses, nor pry into the state of physic which first rendered a corporate body of this kind, with exclusive powers and privileges, necessary. It is evident that the charter empowers them to make bye laws from time to time, "for the good government, superintendance, and *correction*, not only of the College, but of all persons exercising the faculty in the city, or within seven miles thereof." Those who choose to resist the College must pay a fine of five pounds per month during the time they practise within the above boundaries. It appears also that the censors, for the time being, shall have "the superintendance, correction, and government of *all persons exercising the faculty of medicine in any manner* (aliquo modo frequentantium et utentium) in the city and within seven miles thereof, with powers to punish for mal-practice, and with power of superintendance and scrutiny of all medicines and their administration, provided that the punishment should be by fines, amercements, imprisonment, and other reasonable modes." Now, to our apprehension, the above clause completely embraces the means of suppressing quackery in almost all its shapes; and in the name of humanity, we entreat the censors of the College to set about the good work of eradicating these pests of society.* The following quotation will be read with no small interest, and, we should suppose, with some consternation, by the graduates of Edinburgh, Glasgow, &c. now practising in England.—

"The concluding section of this act is important, as it evidently repeals so much of the 3 *H*. 8. as refers to physicians, enacting, 'that 'no person from henceforth be suffered to exercise or practise in

* On proceeding farther, however, we find that this statute or clause is unavailable, for in the case of the College versus Rose, the decision of the House of Lords reversed the sentence of the Court of King's Bench, which had fined the said Mr. Rose in a penalty for administering medicines by his own judgement, and without the advice of a physician. We may here also remark that it does not appear that the College can now amerce an unlicensed practitioner in 5*l*. per month, but sue him at common law for the same. This, of course, would run up the expence to a great amount, as the defendant would be sure to be fined with costs at each suit.

'physic through *England** until such time as he be examined at 'London, by the said President and Three of the said Elects; and 'to have from the said President or Elects Letters Testimonial of their 'approving and examination, except he be a Graduate of Oxford or 'Cambridge, which hath accomplished all Things for his Form 'without any Grace." 17.

Of the history of the litigations between the Fellows and Licentiates of the College we shall take little or no notice. We are sorry the authors of these volumes should have brought up the subject at this time of day, filling whole appendices with trials that ought to be buried in oblivion. We think the republication of these trials, and the various charters and bye laws on which they were founded, a waste of paper, to say the least of it; because, we think it hardly possible that such trials can ever again be instituted. New laws and new modelled charters will doubtless be framed in the progress of human society; but while the present laws and charters exist, we conceive that the old disputes between the fellows and licentiates can never be revived. And here we may perhaps be permitted to make one or two reflections on the subject in question—reflections that have at least the merit of impartiality and independence, for, from particular circumstances, we have nothing to hope and nothing to fear from either of the parties. It is considered a grievance that Edinburgh, the great school of physic, should not have the same privileges that Oxford and Cambridge have, which universities can afford little or no medical instruction. But to us it appears reasonable that an academical education at our English universities should have some privileges over Edinburgh, because a far better preparatory initiation in literature, languages, and science is thereby secured; and it cannot be denied that this early and regular intellectual discipline fits the mind better for scientific pursuits, ceteris paribus, than

* "It is true that the College has no means of punishing the disobedient in the country, because the Statute is not supported by penalties; but it must be remembered that the acting in defiance of a Statute is in itself a misdemeanour. According to the opinion of Chief Justice *Mansfield*, a Doctor's Diploma does not itself entitle the possessor to practise in the country parts (provinces) of England. He must be an Extra-Licentiate of the Royal College of Physicians, or Medical Graduate of an English University. The provincial physician, unless thus protected, is placed under very humiliating circumstances; he is only a doctor by *courtesy*, and therefore cannot claim rank, or defend himself in courts of law. In a cause tried at Stafford before Judge *Mansfield*, a physician who had graduated in Scotland, having been grossly abused in his professional capacity, sued for redress, but could obtain none, because he had not complied with the act of *Henry* the *8th*. *Middleton v. Hughes*. *See Harrison's Address*. 62." 17.

where such discipline has not been put in force. It is vain to object that these seminaries of learning are not schools of medicine. Every one knows, that those who can afford a university education can also afford to cultivate medical science at the proper fountains of instruction. Neither can they come to collegiate honours till they undergo the necessary examinations before a competent tribunal. In short, it is our belief that the faculty of Physicians would be much bettered, if the restrictions on entering that profession were still narrower than they are at preseut, and the facilities less. Such is the influx, in our times, that a considerable proportion of medical society must soon, if it does not now, consist of needy adventurers, who cannot otherwise than deteriorate the profession itself, and lessen its dignity in the eyes of the world. Doubtless every restriction, of the kind now under consideration, must prove a check to merit labouring under the *res angusta domi*, and thus preclude genius from earning the *summi honores* in medicine; but laws must be enacted for the many and not for the few. By these observations we do not mean to advocate the unqualified optimism of the existing laws and regulations as they relate to physicians. We sincerely wish that the invidious distinctions between fellows and licentiates of the London College could be done away with, or lessened; but we see no prospect of such an event, because we cannot deem it either reasonable or prudent that the College should throw open its gates to all who chuse to knock. We do think, however, that the College of Physicians ought, in deference to those universities where many of them have received their own medical education, to admit as licentiates, without examination, those who have regularly graduated there; for example, the diplomatists of Edinburgh and Glasgow. The tests of medical knowledge, that is, the examinations, are stricter at those universities than at the London College, and why therefore subject men twice to the same ordeal? Another remark, which we beg leave to make, is equally applicable to the northern and southern examinations—it refers to the *modus*. If it be desirable (and we think it is) that the candidate should be a classical scholar, why not put it to the proper proof by examining him in some of the classic authors, instead of compelling him to hold a conversation in Latin, by which he can neither prove his latinity nor display his professional knowledge?—We do maintain that this preposterous adherence to antiquated custom defeats, in very many instances, the end and object of the examination itself; because we are certain that many an illiterate dunce has passed muster in consideration of the difficulty of expressing himself in a language of which he knew little, respecting a science of which he knew less. Why, in the name of common sense, should we be obliged to render an account in *Latin* of that which we are *taught* in *English?* Is it not preposterous in a professor to sit three seasons inculcating his precepts in the vulgar tongue, and then to turn short round on his heel, and compel his pupils to render an account of their acquirements in a dead language! In whatever tongue the knowledge is taught by the master, in that tongue should an account of the proficiency be rendered by the pupil. We might appeal to the custom of our continental neighbours, but we rather appeal to common sense, and we challenge the big wigs, North and South of the Tweed, to urge a satisfactory reason *against* the modus which we propose, or *in favour* of that to which they so pertinaciously adhere. We believe the only plea they can assign is *custom;* but even this we impugn; for if they trace things to their origin, they will find that prelections were delivered, and competency was inquired into, in the same language.

2. *College of Surgeons.* We shall not dwell on the "charters, by-laws, ordinances, rules, and constitutions" of this College, since they all end in smoak, "as they do not give any power to restrain and punish ignorant pretenders, who, without the slightest qualification, assume this dangerous and difficult branch of practice, and most especially in the country." To us it does not appear that either college has much to brag of in this respect; for if "Surgeon Goss, &c. stick up their bills under the very noses of Machaon and Podalirius, in Lincoln's-Inn-Fields, Dr. Eady, with equal effrontery and tone of defiance, may inscribe his name, in very legible characters, over the gate of the College of Physicians in Warwick Lane.

3. *Society of Apothecaries.* The laws and regulations of this corporation are now too well known to require any notice in this place. It is evident that this society has more real power than either of the other two. For the College of Surgeons has no power at all over any but those who choose to become its members; and whatever authority the College of Physicians may have over quacks, it is manifest that they do not exercise it. Among the cloud of words by which the charter of the apothecaries is described, we cannot pick out sufficient information to ascertain whether they have any power over the host of Charlatans by which town and country are overrun. Such is the glorious uncertainty of the law!

4. *Actions by Medical Practitioners.* A physician cannot recover fees by an action—not even his travelling expences—unless a promise is made, or a bond or bill is executed. A surgeon, of course, can recover medical attendance. "All physicians may practise surgery, though they (surgeons) may not encroach in physic." This is a specimen of the concordance between law and fact. We appeal to the reader whether the reverse of the above rule does not obtain in actual practice? The truth is, that all surgeons may practise physic, while scarcely any physician dare practise surgery, even in provincial towns. The M.D.'s of Edinburgh and Glasgow, who swarm through Scotland, practise as surgeons, and not as physicians in general: and as to England, there is scarcely such a thing as a physician practising surgery and physic at the same time.

5. *Actions against Medical Practitioners.* If the following piece of *law* can be construed into *sense* by any of our readers, we congratulate them on the brightness of their intellect, for to us the passage is an incomprehensible paradox.

"If a physician, surgeon, apothecary, or other medical practitioner, undertakes the cure of any wound or disease, and by neglect or ignorance the party is not cured, or suffers materially in his health, such medical attendant is liable to damages in an action of trespass on the case: but the person must be a *common surgeon*, or one who makes public profession of such business, as surgeon, apothecary, &c. for otherwise it was the plaintiff's own folly to trust to an unskilful person, unless such person *expressly* undertook the cure, and then the action may be maintained against him also." 80.

In the first part of the passage, physician, surgeon, and apothecary are liable to action—in the middle, it must be a "common surgeon"—and towards the end of the passage, it must be some person who *expressly* undertakes the cure, when he *also* may be prosecuted!! This is surely throwing dust into our eyes, instead of clearing our optics.

It appears that "any deviation from the established mode of practice shall be deemed sufficient to charge the surgeon &c. in case of any injury arising to the patient." A Mr. Slater recovered 500 pounds damages from Messrs. Baker and Stapleton, "for unskilfully disuniting the callous (callus) of the plaintiff's leg after it was set, which it appears was done for the purpose of trying a new instrument." A surgeon is held responsible for the negligence and ignorance of his apprentice or servant. Though the cases on record are all surgical, yet our authors think that physicians are equally amenable—but, happily for them, it is not quite so easy to demonstrate a wrong dose of physic in the stomach as an elbow in the os femoris after a fracture.

6. *Midwifery.* Under this head we were amused with the probable reasons why bishops formerly licensed midwives—namely, that they might be proved capable of repeating the form of baptism over a child's head, in case the body followed rather tardily. We confess that we are unable to sympathize in the gravity of the following sentiments.

"Surely, if the baptism of a child by a lay person is good, and the body cannot have Christian burial without it, there is nothing senseless or irreligious, and we will venture to add nothing morally or legally wrong, in the performance of this provisional ceremony. If there were no other object than to satisfy the anxiety of the mother at a moment when the calmness of her feelings is vitally important, it ought not to be omitted whenever the danger of the child and the absence of a priest appear to render it necessary." 83.

We are doubtful whether, even amongst the most stanch Catholics of these realms, there be such anxiety about baptismal aspersions of half-born infants, as to render it necessary for the doctor to stand divine at man's entrance into the world, in the same way as Blackstone has recommended him to act the lawyer while making his exit. Be this as it may, all we can glean from the present section of the work is, that "there is some probability that both the College of Physicians and the College of Surgeons will decline in future interference with this branch," the obstetrical. If this be so, and no tribunal be appointed to examine and license accoucheurs, an important branch of medical science will be left at the mercy of every ignorant pretender.

7. *Preservation of Public Health.* In this country, so famous for its laws, there is no separate legal code for public hygiene. Actual nuisances are actionable at the suit of the parties aggrieved; but, excepting quarantine, the executive government takes little or no part in securing the bodily health of its subjects. Our authors remark, that the habits of order and cleanliness in the inhabitants, and the "general salubrity of our climate" may have rendered such care less necessary, while our spirit of liberty and independence might have resisted the encroachments on domestic privacy, and the perpetual intrusion of local authorities to which our continental neighbours are subjected. It is needless to pry into old and obsolete laws against contagions, lepers, lazars, plague-sores, &c. but it may be proper to bear in mind that it is still an indictable offence for any person to pass through the streets with small-pox on him. Our authors are surprised that no legislative measures have been instituted to check the propagation of venereal diseases, which they consider, however, as on the decline. They suggest that a surgeon should be attached to each police office, and that the magistrates, in clearing the streets for the night, should order the detention of those whose liberty might prove dangerous to the unwary. They also hint, that some regulations should be formed respecting manufactories and schools, when contagious or epidemic diseases are prevalent, in order that the infected might be speedily removed from among those who are well. Such regulations would be extremely difficult of formation—or, at least, of execution, for obvious reasons.

Our authors very properly animadvert on the impolicy of such taxes as have a tendency to deprive the lower orders of those articles that are necessary to health—for instance, the tax on salt, an indispensible part of their diet. The importance of cleanliness is also properly insisted on, as well as the deleterious influence of stagnant waters. The slaughtering of cattle is another nuisance which imperiously demands the interference of the legislature; and still more the practice of burying the dead in churches, church-yards, and cemeteries situated in the very centres of our most populous cities. The cemeteries of this metropolis are so crowded that it becomes much more difficult to find room for the dead than for the living; and it is notorious that there are many church-yards in which the soil has been raised several feet above the level of the adjoining street, by the accumulated remains of mortality. There are others in which the ground is actually bored before a grave is opened. The most offensive, if not deleterious instance of this kind, occurs in the Irish metropolis.

"There is a burial ground at the back of Kilmainham Hospital, (and consequently under the immediate view of the Commander and Adjutant-General of the Forces,) so disproportioned to the number interred in it, that the older coffins are frequently broken, and the undecomposed limbs constantly thrown on the surface, to make room for new tenants of this human soil; yet, after heavy showers, the earth being washed away, the lids of coffins may be plainly discerned, so slight is the covering which can be afforded them. Immediately below the rising ground on which this cemetry is situated, are the Island Bridge Barracks for the Artillery, the wells of which must of necessity be filled with the filtrations from the putrid mass above them. One at least of the principal Tanks at Gibraltar was similarly situated. The present Lieutenant Governor, Sir George *Don*, among the numerous improvements in the regulation of cleanliness and ventilation which he has introduced on the rock, has converted the burial ground into a public garden; to this, among his other measures, the garrison may owe some future exemptions from the diseases which have so often afflicted them." 94.

Although our authors agree with Dr. Bancroft, that putrifying human bodies are not capable of generating the specific contagious matter of plague, typhus, or any true pestilential fever, yet they cannot but believe that, in the decomposition of the human body, various noxious principles are developed injurious to life. They republish the old account of the exhumations from the cemeteries of the St. Innocents at Paris, from which undertaking it does not appear that "epidemic evils" were experienced; but, as they properly observe, the greater part of these mortal remains had been converted into a harmless substance resembling spermaceti. Had this change not taken place, it is probable that worse consequences would have ensued from the exposure of this horrible accumulation. But, although no "epidemic evils" occurred, (as indeed there was no probability,) individual examples were not wanting to prove the dangerous tendency of the mephitic vapours emitted. Grave-diggers were thrown down suddenly, and deprived of sense and motion, on breaking open with their spades the abdominal viscera of the corses—and in a wider circle of dilution these vapours induced nausea, loss of appetite, debility, and tremors.

"If farther evidence be required upon this subject, we have only to direct the reader's attention to the effects occasioned by opening the graves in St. Dennis, and to which no allusion is made by *Dr. Bancroft:* the National Convention in the year 1793, in the true spirit of revolutionary ferocity, passed a decree upon the motion of *Barrère*, that the monuments of the kings in this, as well as in all other places throughout France, should be destroyed. In carrying this decree into effect, the bodies of many of the latter Bourbons were found in a state of decomposition, and when the coffins were opened they are said to have emitted a thick black vapour, which, although vinegar and gunpowder were burnt to prevent ill consequences, affected the wretches employed in this inhuman work with fevers and diarrhœas: so again, when the ground of the church of St. Benoit was dug up a few years ago, a nauseous vapour was emitted, and several of the neighbours were affected by it." 97.

We cannot participate in the sceptical hardihood of our authors when they deny "that putrid emanations from the bodies of persons who have died of a pestilential disorder are capable of re-exciting the disease." We refer our authors to what Dr. Jackson (who will not be suspected of entertaining very credulous opinions on contagion) experienced in his own person when opening the bodies of men who had died of the epidemic fever at Cadiz. And as our present authors adhere to the doctrine of typhus being generated from a specific miasm, how does the above scepticism quadrate with the following *analogous* fact?

"We ought not, however, to omit to state, that instances are on record, where the small-pox has suddenly appeared in a village, after opening the grave of a person who had a few months before fell a sacrifice to that disorder." 98.

It is computed that the decomposition of the soft parts is not terminated until the expiration of three years, in graves of four feet deep—or four years where their depth is six feet, varying, however, with the nature of the soil.

The cleansing of privies deserves some notice in this place. It has long been admitted, that a deleterious effluvium (sulphuretted hydrogen gas, sometimes in combination with ammonia*) issues from these receptacles of human ordure, which has been known to occasion ophthalmia, diarrhœa, dysentery, and sometimes sudden death, when the emanations were much concentrated.

" *M. Dupuytren* has given us many particulars respecting this affection; sometimes the patients are strongly asphyxied, and death takes place in a very short period; at others, the symptoms are less intense, and if the patients be carried into the open air, after a short interval, they make deep inspirations, and the breathing is gradually restored, although it continues laborious: the motion of the heart becomes perceptible, nevertheless the pulse is weak and small; the digestive and loco-motive apparatus have lost their contractile force; the functions of the brain are suspended; and if the patient finally recovers, he is a long time in re-establishing his strength. An emetic appears to be the remedy upon which the *nightmen* rely for relief." 101.

Explosions of hydrogen gas are not very uncommon in privies—especially in the Parisian ones, where cleanliness is not the order of the day, although a royal ordonnance on this subject contains thirty-four clauses or articles, enjoining most expensive and troublesome buildings or repairs of privies.

" And upon this occasion is is impossible to withhold the expression of those feelings of national pride and exultation which the contemplation of this subject must afford us; we have in our metropolis no less than 200,000 privies, of which 10,000 only are water closets. In Paris the number does not exceed 70,000, and yet with all the cumbrous enactments which that government has passed for their regulation, how far inferior they are in cleanliness, and how far greater are the effects of their effluvia, when compared with similar establishments in our city. The truth is, that the most elaborate system of medical police will never be so effective as the spirit of cleanliness, which is so characteristic of this great and free people;

---

* M. Dupuytren thinks he has satisfactorily proved that chlorine, by decomposing it, is its true antidote, by which hydrochloric acid (muriatic) is produced, and sulphur deposited.

and in this truth, so forcibly illustrated by the subject under discussion, we are to seek for the real explanation of that fact which has been so frequently commented upon by medical writers—THE APPARENT INDIFFERENCE OF OUR GOVERNMENT TO THE SUBJECT OF PUBLIC HEALTH." 102.

It is well remarked by our authors that the commissioners of sewers, though not engaged, like the œdiles of ancient Rome, in constructing magnificent aqueducts, are yet occupied in the far more stupendous and wonderful works which extend beneath the foundation of our mighty city, and which dispense to its inhabitants the essential requisites for comfort, cleanliness, and health.

8. *Quarantine Police.* It is hardly to be expected that the authors of the work before us should throw any new light on the long litigated questions respecting contagion, epidemics, endemics, infection, pestilence, &c. They justly remark, what indeed we have often remarked ourselves, that it is ridiculous to squabble about the etymoligies of the words contagion and infection, as their import is not necessarily to be deduced from their derivation, but from their definition, or rather from established usage. But the fact is, that the distinction which the etymological physician would establish between the above terms is not accurate, for in every case of infection there is actual contact of morbific matter, whether visible or not; while some diseases, as the small pox, are communicated both by palpable matter, and by imperceptible effluvia.

Our authors now come to the momentous question, "*whether epidemical disease be ever propagated by contagion?*" In respect to plague, this is their creed. "We may state, in general terms, that the concurrent testimony of different ages and countries sanctions the opinion that plague arises from specific contagion—is communicated *immediately* by contact, or *mediately* by the agency of infected goods;—and that its progress may be arrested by a vigilant system of police, cutting off every communication between the healthy and the infected." Our authors pass some severe censures on the writings of Dr. Maclean—writings which we have long looked upon as ravings, and consequently unworthy of notice. On the principal questions connected with epidemic diseases our authors entertain nearly the same sentiments which have been long maintained in this Journal, and with which our readers are sufficiently familiar. It is hardly necessary to remark that the authors of the work under review are implicit believers, not only in the contagion of plague, (in which we agree with them,) but in the omnipotence of the quarantine system, if rigorously executed. We have very great doubts, however, respecting the apprehended danger of importing the plague into this country, if the quarantine laws were abolished. We do not believe England to be the soil on which the fomites of plague can grow; and for this reason, that, if our climate and manners were compatible with plague, it would have made its way hither long ago, in spite of vinegar and fumigations. The following is certainly no very forcible argument in favour of the *success* of quarantine laws in restraining the encroachments of plague upon the harbours and coasts of Italy, France, and Spain. Speaking of the quarantine officers of those countries, our authors observe—

"Occasionally indeed the courtesy of these gentlemen will deem a governor or wealthy noble to be incapable of communicating infection, though from the most suspected port, while a whole fleet of merchantmen, arriving with clean bills from the Atlantic, will be detained for some weeks, *ex abundanti cautela*, without admission to *pratique*." 130.

We are not advocates, however, for rash experiments, and therefore wish not for a sudden abrogation, though we hope there will be a gradual amelioration of the restrictions now imposed on commerce and communication. This appears to be the sentiment of our authors also. "At the same time we are ready to admit, with a periodical writer, that quarantine regulations might be amended, and rendered less inconvenient to commerce. They might, for instance, be modified as to the required period of segregation." 137.

9. *Medical Police.* With the exception of quarantine laws, laws of nuisance, and visitations of mad-houses, it can hardly be said that there is such a thing as medical police in this country. Our authors justly wonder why the sale of factitious, impure, spoilt, or deleterious drugs, should be guarded against *only* in the metropolis, (by the censors of the College,) while the provincial towns are left to their fate. To obviate the fatal effects arising from the exhibition of arsenic, our authors suggest that it should only be sold when mixed up with tallow, as the excuse for purchasing it is always the destruction of vermin. Speaking of the dreadful mistakes which every day occur from taking oxalic acid in lieu of Epsom salts, our authors observe—

"*Oxalic acid*, to which so many deaths have been lately attributed, may serve as an instance; in its external characters it bears such a resemblance to those of common *Epsom salts*, as readily to deceive the ordinary observer; and as both substances very frequently become articles of retail custom, they are usually kept ready for sale, in parcels of an ounce each, a practice which renders a careless substitution an error of common occurrence; the employment of a particularly coloured paper, that of *yellow* for instance, if used universally as a wrapper for poisonous articles, upon which also the word *poison*, or *dangerous*, might be legibly printed, would to a certain degree guarantee the safety of the purchaser; but as danger might notwithstanding be apprehended in the night, a paper of a distinct texture might afford additional security; the peculiar roughness of the Dutch filtering paper, which is manufactured from woollen, would answer such a purpose. The labels of phials should in this particular correspond with the wrappers of dry substances; if the distinction were once generally adopted by the various dealers, it would soon become notorious to indifferent individuals, and many fatal accidents might be prevented, without the aid of legislative enactment.* 142.

10. *Bills of Mortality.* That much error and confusion must creep into these records from the absurd manner in which the investigation is conducted, will appear from the following extract:—

"The churchwardens of each parish within the bills of mortality, appoint two old women to the office of *Searchers*, who, on hearing the knell for the dead, repair to the sexton of the parish, to learn the name and residence of the deceased. They demand admittance into the house to examine the body, in order that they may see that there is nothing suspicious about it, and *judge* of what disease the person died, which they report to the parish clerk. The regular charge for the performance of this office is *fourpence* to each *searcher;* but if an extra gratuity be tendered, they seldom trouble the domestics with any examination." 144.

We agree with Dr. Burrows in thinking that this office, as at present filled, should be entirely abolished, and that the attestation of a properly qualified medical practitioner, upon actual knowledge of the disease or enquiry and examination of the body, should be substituted. Were the bills of mortality properly kept, we should derive from them important information on many dubious points, as the rise of many diseases and their affinity to each other—the increment, decrement, and cessation of epidemic and contagious diseases—the comparative salubrity of different countries, places, climates, and seasons—the influence of particular trades and manufactures on the human constitution, &c. Notwithstanding all their defects they are still very useful, and they establish, beyond all doubt, the gratifying fact of the superior salubrity of London, with all its increase of population,

* "A bill was recently introduced in the House of Commons on this subject, but did not pass into a law."

in the present day, to what it was during the seventeenth century, when the deaths exceeded the births by more than one half of the whole number; while in the present age the sum total of births exceeds that of deaths. The same improvements have taken place in the provinces, and the value of human life is progressively increasing throughout Great Britain.

Here ends the first part of the work before us. We have endeavoured to cull out the most interesting particulars; but we fear our readers will be ready to say that we have hardly succeeded in keeping them awake during the perusal. It is not, however, the fault of the authors or of the reviewers, but of the nature of the subjects discussed.

11. *Medical Evidence.* Much of the matter introduced by our authors, under this head, applies to evidence in general, as the penalties for disobedience, the expences allowed, &c. With these we need not meddle, although a list of works, on the law of evidence, is recommended to the perusal of the medical practitioner, that would occupy him some months, if not years, to study! For own parts, we swear never to peruse one of them.

We shall glance, however, at some points of information laid down in the volumes before us. It appears that a medical man is not justifiable in withholding from the Court any information which has come to his knowledge in the way of his profession and under strict secresy. The secrets of the prison-house must come out. When we consider the difficulty, or rather impossibility of bringing many medical men into the same way of thinking on abstruse points of physiology or pathology, we can hardly wonder at the constant clashing of medical evidence in courts of justice. Even in chemistry, which now assumes the character of an exact science, we have lately seen the most ludicrous specimens of conflicting testimony in the cases of Severn, King, &c. against several fire insurance offices.

"The causes were conducted by professional men of the first eminence, the Judge who presided well known for his love of science, and from having attained more knowledge in several branches of natural philosophy, than can usually be acquired by those whose time is engrossed by severer studies; the witnesses were among the best Chemists of the day, yet the question (simple as it might at first appear) whether oil or sugar at certain temperatures, and under certain circumstainces, should be considered the more inflammable substance, occupied three days on the first and six days on the second trial. Notwithstanding which, a third trial took place involving the same question, and controversial pamphlets were published on both sides on the nature and supposed contradictions of the evidence." 160.

As to the mode in which a medical witness should deliver his evidence, there is much difference of opinion. While some recommend their pupils or readers to open at once the stores of their reasoning and information, others, fearing the effect which cross examinations may have on nervous or embarrassed witnesses, advise that no more shall be disclosed than categorically meets the question of the counsel:—

"And to this we incline, with this difference, that as we should deem too costive a retention of the truth as blamable as the flow of garrulity with which we have sometimes seen a court overwhelmed, we recommend the witness to steer a middle course, first answering patiently, distinctly, and tersely, the questions put by the Counsel on both sides, the Court and the jury; and if none of these elicit the whole truth, and any material point remains to be disclosed, the presiding judge will always admit and gratefully receive the additions or explanations which may be necessary to the ends of justice." 164.

It may be necessary to bear in mind that notes, if taken on the spot, or immediately after a transaction, may be used by the witness to refresh his memory. But these notes must be original, not copies.

A medical witness (for instance when he goes out professionally with duellists) need not answer to any thing that will subject himself to a criminal prosecution. Mr. Pettigrew, of Spring Gardens, refused to answer questions in the case of Mr. Patmore and Mr. Scott, when the latter was shot in a duel.

12. *Marriage.* We must pass over all the legal portion of this section, as not at all interesting, as far as we can see, to medical men more than to other men. Under the head of AGES as connected with marriage, divorce, &c. much tiresome and tedious matter crowds upon us; and to shew that this is not a captious and ill-grounded remark, we shall subjoin a specimen for the perusal of our readers.

"INFANCY—*Infantia*—(from *Infari*, not able to speak) commences at birth, and terminates at the *seventh* year. The signs by which the age of an infant may be computed, are derived from its moral as well as physical characters; and as circumstances connected with medico-judicial inquiries may render the problem of importance, we shall proceed to offer some data that may assist its solution. The feebleness and size of the infant; its epidermis yet reddish, and wrinkled; its face covered with down; its head soft, and the *fontanelles* greatly extended; the eye but little sensible to light, and lastly the appearance of the navel, are circumstances which will at once lead the medical practitioner to the conclusion of its not being many days old; while its smiles and tears, its upright posture in the nurses arms, the thickness and whiteness of the skin, the plumpness of its thighs and buttocks, the eagerness with which its eyes seek and follow brilliant objects, its agitation on the occurrence of noisy sounds, and its eager desire for the breast, are occurrences which will, according to the force and degree of each, announce the child's progress towards the *third*, *fourth*, or *fifth* month. The pleasure which it testifies at the sight of its nurse, its jealousies, and other passions, the habit of carrying its fingers and different objects to its mouth, the facility and pleasure with which it chews bread, and the copious discharge of saliva, announce the approach of dentition, and assure us that the infant must be in its seventh month. The progress of dentition will at this period afford some farther data; towards the end of the *seventh* month the middle Incisor teeth of the inferior jaw perforate the texture of the gums; and soon afterwards the corresponding Incisors of the upper maxilla make their appearance; then the lateral Incisors of the inferior, and subsequently those of the superior jaw; about the *twelfth* or *fourteenth* month, sometimes sooner, the first of the Molares of the under, then the corresponding teeth of the upper jaw appear; the four Cuspidati are usually protruded through the gum the last; thus the Cuspidati and the second Molares will sometimes appear at the same time, and this is usually between the *twentieth* and *twenty-fifth* month; so that at, or soon after *two years* of age, the twenty temporary or *milk* teeth are to be found in situ. It must however be remembered that the formation and appearance of the *milk* teeth are subject to considerable variety, and there are some examples on record, though very uncommon, of children born with two Incisors in the upper maxilla, but such teeth have been found to be imperfect in their structure, and without fangs, and they have consequently soon been detached; in other cases, children, although enjoying perfect health, have not cut a single tooth until the end of their second year. Nor are the other signs to which we have alluded, as affording indications of the age, to be considered as immutable; the infant may have been more or less retarded, or accelerated in its march of developement by its state of health and vigour, and it deserves remark, that scrophulous and *rickety* children very commonly present an aspect of intellectual precocity, by no means commensurate with their age; and hence the popular notion has arisen, that very intelligent children rarely continue to live. The fact of this premature expansion of the mind is too apparent to be doubted; but philosophers have endeavoured to explain it upon very different principles; the physiologist has sought the cause from some peculiarity in the organization of the body, while the moralist has attempted to account for it by supposing that in consequence of the inability of these subjects to partake of the sports and exercises suitable to their years, they necessarily enjoy more of the instructive society of their parents and preceptors." 184.

Pueritia, or boy-hood,—adolescence, or puberty,—juventus, or youth—ætas virilis, or manhood—senectus, or old age, and decrepitudo, or

"Last scene of all that ends this strange eventful history,"

are each handled with equal or more minuteness. We shall say but a few words on these subjects as they rise in succession.

Pueritia commences with the shedding of the temporary or milk teeth, about the seventh year, and ends at puberty, the 14th or 15th year in males, and 12th or 13th in females. At this important epoch the powers of the system are directed into other channels besides the mere growth of the body. In the male, the beard sprouts—the voice grows rough, the aperture of the glottis augmenting in the stronger sex in the proportion of 5 to 10—in the weaker only in the proportion of 5 to 7—the generative functions are established—the whole volume of the body augments and assumes a character so decidedly masculine as at once to proclaim the sex of the individual. The mental phenomena correspond with the corporeal. The mind acquires tone—the manners and habits become more manly. At this critical period diseases often commence or disappear.—Hereditary mania, phthisis, scrofula will generally lie dormant till this epoch, and then declare themselves. It is well known that, in the female, the mammæ enlarge, and the catamenial discharge appears at this period. It is quite useless to digress to the histories of those rare cases of precosity in both sexes which, being exceptions to the general laws of nature, are mere matters of curiosity, and useful only to swell the pages of an ephemeral print. The phenomena of puberty depend, of course, on the developement of the generative organs—but not of all parts of these alike. The testicles and ovaria seem to be the important parts. Females have grown up with the characters of perfection, where the uterus was wanting ; but not so when the ovaria were defective.

Youth succeeds to adolescence, and is tacitly allowed by the laws as the period best adapted for the vigorous discharge of public duties. "It is the age at which the greatest enterprizes have been achieved, and the most brilliant efforts of human genius fulfilled; the developement of the body having been accomplished, its powers are expanded in the production and support of intellectual energies." In this period the action of the arterial system may be said to predominate over every other, and hence the diseases to which man is exposed in this stage of his existence are of an acute and inflammatory character.

*Manhood.* Youth passes into manhood by such insensible gradations, that it has been considered as only a continuation of the same stage of perennity. Hippocrates and Galen compare the youth to summer, and manhood to autumn, thus insinuating that, if the one be less fervent, it is also more mature than the other. In the latter, what is lost in glow of imagination is gained in maturity of judgement. The arterial system no longer predominates over every other—its energies are reduced, and a juster equipoise is established. The diseases of man now assume a different aspect, and maladies of a chronic nature prevail. Thus, while in the apparent plenitude of our existence, we are fast journeying to our final goal. Man, in fact, never stands still. In vain shall we attempt to cast anchor in the stream of life. It will alike carry away those who struggle against it, and those who yield quietly to the force of the current. Any panacea to renovate the body is as chimerical as the "fountain of youth" is fabulous.

Nascentes morimur, finisque ab origine pendet!

*Senectus—Old Age.* The bony framework has now become more solid—the vascular system abridged in the extent and subtlety of its ramifications—the muscles less irritable—their adepose matter less plentiful—the whole volume diminished —in short, it is in this age that we shift into the "lean and slippered pantaloon." The senses lose their acuteness, the heart its force. Venous plethora prevails—the blood vessels are liable to ossific depositions, whence apoplexy and various affections of the heart and other organs arise. The faculty of reproducing the species ceases long before the natural termination of life, though this failure is far more uncertain than the commencement of the function.

" Decrepitudo—*Advanced Age.* At length the limbs fail under the burthen which for so many years they had sustained with ease; the exterior muscles gradually return to that state of debility in which they were during infancy, and being unable to sustain a continued state of contraction, relieve themselves by alternate intervals of relaxation, from which arise the tremors* so characteristic of old persons; upon the same principle is to be explained the *Vacillatio Senilis,* (see-saw) for by these motions the muscles which preserve the perpendicularity of the body, are alternately quiescent, and exerted; and are thus less liable to fatigue or exhaustion, The teeth having successively dropped out of their sockets, the alveolar processes are absorbed, and the projection of the lower beyond the upper jaw, imparts a very peculiar physiognomy to the countenance.

" Last scene of all,
" That ends this strange eventful history,
" Is second childishness, and mere oblivion,
" Sans teeth, sans eyes, sans taste, sans everything." 196.

---

* " This is erroneously supposed to be paralytic, they evidently originate, says Dr. Darwin, from the too quick exhaustion of the lessened quantity of the spirit of animation, for they only exist when the affected

And now we hear (or fancy we hear) some of our readers ask what has all this sentimental physiology to do with forensic medicine?—After shifting our wig, rubbing our eyes, taking snuff, and looking very grave, we are constrained to answer that—we do not know!—All the excuse we can make is, that we find these things recorded in a work on medical jurisprudence by doctors and lawyers of rank and reputation. Certes, we might just as well have been amusing ourselves and our readers with dissertations on the circulation of the blood, muscular motion, or nervous influence; but such as we find in books, ex professo, such must we give unto thee, gentle reader.

13. *Impotence and Sterility* follow the subject of *marriage*, in the work before us—which must be a great consolation to the disciples of Malthus—provided they are as consecutive in the works of Nature.

The causes of impotence may be absolute or relative—functional or organic—physical or moral—temporary or permanent.

*Organic Causes in Males.* There was a period in the history of physiology, observe our authors, "when the testicles were not considered essential to virility." The Stagirite, who set himself up as a judge in physiology as well as poetry, asserted that the testicles merely served "as weights to hinder the spermatic vessels from being folded up." We think it would have been almost as good a comparison, or rather conjecture, if he had likened them to the pendulum or the weights of a clock, which regulate the movements of the gnomon or index above. Every one knows that these bodies descend in general from the abdomen to the scrotum. In some they do not come down. What then? Foderé states that such persons have even been remarkable for their vigour, "for these organs, says he, appear to derive greater power of secretion from the warm bath in which they lie, than when they have descended into their natural situation." John Hunter entertains a different opinion. He believes that when both testicles remain through life in the belly, they are exceedingly imperfect, and incapable of performing their natural functions. In this opinion Zacchias and Riolan concur; while the late Mr. Wilson stated, that he was acquainted with one case that confirmed, and another that refuted, this last opinion;—and

---

muscles are excited into action, as in lifting a glass to the mouth, or in writing, or in keeping the body upright, and cease again, when no voluntary exertion is attempted."

this is probably the true state of the question. Each case must rest on its individual merits.

It does not appear that two testicles are essentially necessary for procreation, no more than two eyes for sight, though the Parliament of Paris declared the matrimonial contract null where one testicle was absent! That Parliament has since had other fish to fry. The testicles are liable, of course, to many diseases that destroy their texture or function, as scirrhus, cancer, &c. M. Larrey has described an atrophy of the testicles which affected the French soldiers returned from Egypt, by which these organs became soft to the touch, and gradually diminished in size, without any pain. It is curious, too, that persons afflicted with elephantiasis lose the venereal appetite, and suffer a wasting of the genitals. We cannot dwell on the various organic and mechanical defects to which the parts of generation in both sexes are liable—they will be readily appreciable by men possessed of proper anatomical and physiological knowledge.

Sterility in women is much more frequent than impotence in men. The causes are numerous, as absence of the uterus, imperforate uterus, polypus in utero, ovaria wanting or diseased, &c. Most of these causes are not only irremediable, but incapable of being detected during life. There are many functional causes of sterility in women, as constitutional debility, leucorrhœa, irregularity of the menses.

"Women who are very corpulent are often barren, for their corpulence either exists as a mark of weakness of the system, or it depends upon a want of activity in the ovaria; thus, spayed or castrated animals generally become fat." 215.

The cause of general barrenness in prostitutes has never been satisfactorily accounted for. "Exhaustion of the uterine system occasioned by frequent and promiscous intercourse with the other sex," is what we cannot easily comprehend. Most of the lower classes when they first go on the town are healthy, vigorous, and cannot become exhausted all at once —yet it rarely happens that they become pregnant, even in the earliest period of their prostitution. We imagine it is more from mental than corporeal causes that this exemption results.

14. *Legitimacy of Children.* On this point, the laws of happy old England are very indulgent to the child—for provided it be *born* in wedlock, it is quite immaterial where it was *begotten!* If indeed it can be proved that the husband was absent *from the kingdom*, nine months or more, then the issue, during that period, shall be bastards. "But it was held that if the husband was in England during any part of the time between the conception and the birth (without reference to the physiological impossibility of the fact) the child would be deemed legitimate."

"But none can be legitimate who are born out of wedlock; in which our law differs materially from the Roman or Canon law, and it is somewhat singular that the celebrated "*quod nolunt Leges Angliæ mutare*" of the Barons, at the Parliament of Merton, in the 20th of *Henry* the 3d, should have been induced by an attempt on the part of the bishops, (*omnes episcopi magnates*) to introduce this novelty,—that children born before marriage should be legitimised by the subsequent performance of the ceremony between their reputed parent." 218.

This conduct of the Bishops is to be entirely imputed, of course, to their being imbued with more Christian charity towards the frailties of human nature, than were the ferocious and uncultivated Barons. Our readers are already acquainted with the importance which is attached by our authors to a knowledge of "Tenant to the Courtesy," by medical practitioners. This then is the law.

"Tenant by the courtesie of England is where a man taketh a "wife seized in fee simple or in fee taile general, or seized as heir "in taile especial, and hath issue by the same wife, male or female, "born alive (*oyes ou vife*), albeit the issue after dieth or liveth, yet "if the wife dies the husband shall hold the land during his life by "the law of England, and he is called tenant by the courtesie of "England, because is this used in no other realme but in England "onely. And some have said that he shall not be tenant to the "courtesie unless the childe which he hath by his wife be heard "crie; for by the crie it proved that the child was borne alive. "Therefore Quære." 225.

As considerable property may depend on the question whether a child be born dead or alive, it seems desirable that notes should be taken by the accoucheur.

15. *Monsters and Hermaphrodites*. These cannot inherit according to law. There is little occasion, however, for law in these cases, since very few monsters who have materially deviated from the human form, have long survived their birth.

16. *Physiological Illustrations*; and first, of conception, or utero-gestation. Our authors have endeavoured to steer as clear as possible of the mazes of ingenious theories and conjectures that have been, from time to time, emitted from the brains of anatomists, physiologists, and physicians, respecting this mysterious process, confining themselves to, and resting on, the sensible phenomena attending it.—

We can pretty confidently say, that the series of changes constituting conception and gestation, commence in the ovaries, and not in the uterus, as was formerly supposed. One or more vesicles become loosened from their connexion, by the nisus of coition, and grasped by the fimbriæ of the fallopian tube, through which they descend into the uterus, leaving a spot in the ovarium, which, when examined after death, exhibits a slightly lacerated appearance, and is called a *corpus luteum*. While these actions are proceeding, the uterus undergoes certain changes. It becomes more vascular—its internal lining softer, and a white mucus is secreted which gets organized, and forms a lining to the whole of the cavity of the uterus, except the openings leading to the fallopian tube. It is termed the decidua, or the decidua reflexa. The time required for the descent of the ovum into the uterus is doubtful. Haller has never been able to distinguish it before the seventeenth day. As the mouth of the pregnant uterus is sealed up with gelatinous matter from the time of conception, the catamenia cannot, our authors believe, afterwards appear. Burns and Denman are of this opinion, the former conceiving that the discharges which have been mistaken for catamenia during utero-gestation, were only secretions (different in nature from the menses) furnished by the vessels of the vagina. We need not dwell on the early symptoms of pregnancy, constitutional or general, as they are not taken into account in a court of law, since the plea of pregnancy will not avail until it is so far advanced as to render our investigations easy and satisfactory. We wonder, therefore, that our authors have filled up so much space with the common elementary matter to be found in every book on midwifery and physiology.

It is about the fourth month after conception that we are enabled to form a pretty good guess, whether the female be pregnant or not. Our authors say that at this period we are able "to place the fact beyond the reach of conjecture." But surely this is too confident language. We have seen cases where the most experienced accoucheurs were unable, even at a later period than four months, to ascertain the fact of pregnancy. Before this period, observe our authors, we cannot determine the question by any examination *per vaginam*, for the fundus uteri is the portion first distended after conception, while the cervix, the only part we can feel, does not begin to shorten to any appreciable extent, before the period just stated.

"The following method of examining the uterus, in order to ascertain whether it be gravid, is proposed by *Torlosa*, and is well calculated to accomplish the object. The woman, being fasting, and her bowels and bladder having been previously evacuated, should be directed to lie down, with the loins low, and with the head and buttocks elevated; the knees are then to be raised and bent, so as to bring the thighs to the belly, and the heels to the buttocks, by which position the abdominal integuments will be relaxed; the midwife is then to place the hand upon the epigastric region in such a manner that the little finger may rest on the pubes, and the thumb on the navel, and ordering the woman to breathe hard, he must press the belly gently during the expiration: if the uterus be gravid, and is more than three months advanced, he will at this moment feel above the pubes an equal, hard, globular body; and if the same examination be made after the fifth month of gestation, he will probably feel at the same time the motions of the fœtus; but, in cases where no tumour can be distinctly felt, the operator must be very careful not to be deceived by motion, for the action of flatus may mislead him, and even where an obvious enlargement exists, the pulsations of the aorta may lead to it a deceptive motion; this is particularly striking where the ovarium is extensively diseased, or the uterus is distended with tumours, an occurrence which has not unfrequently induced the patient to consider herself pregnant; in such a case the ovarium may be felt through the parietes of the abdomen, sometimes pretty high, like the uterus, or like a prominent part of a child, but the round and circumscribed nature of the tumour can never deceive an experienced midwife. *Avenzoar*, however, has left a confession that he was deceived about his own wife, whom he had treated as dropsical, though she had passed her fourth month of pregnancy. We ought also to state that dropsy and utero-gestation may be co-existent, and there are unfortunate cases on record where, on such occasions, women have been sacrificed by the mistaken applications of the trocar.

"In order to ascertain the exact state of the *os uteri*, an examination must be made *per vaginam*, which may be conveniently effected while the woman remains in the same position, by introducing the fore and middle fingers of the right hand. For the first three months the *os tincæ* feels smooth and even, and its orifice is nearly as small as in the virgin state; when any difference can be perceived, it will consist in the increased length of the projecting tubercle of the uterus, and the shortening of the vagina from the descent of the fundus-uteri through the pelvis: this change in the position of the uterus, by which the projecting tubercle appears to be lengthened, and the vagina proportionally shortened, chiefly happens from the third to the fifth month." 238.

Now we appeal to our obstetric brethren whether the marks described in the foregoing examination enable us "to place the fact beyond conjecture?" Nay, we hesitate not to affirm that the first, or external part of the examination—namely, the guaging by thumb and little finger from navel to pubes, as proposed by Tortosa, is a piece of legerdemain, on which no dependance can be confidently placed. And as for the alteration about the os tincæ, we believe few accoucheurs would risk a diagnosis on such equivocal criteria.

These criteria become less equivocal, no doubt, as utero-gestation advances. About the 16th or 18th week the phenomenon of quickening takes place—the laws of England still sanctioning the false hypothesis that it is at this moment the fœtus receives the essence of vitality. The plea of pregnancy in bar of punishment, and the degree of criminality in cases of *abortus procuratus* hinge on this said hypothesis. Yet it is difficult to say, why a fœtus of 3½ months should not be entitled to the same protection of the law as one of 4½ or 5 months. "Et voila," as Mahon says, "le tort immense que font quelquefois les systême et les opinions scholastiques."

The following explanation of the peculiar sensation termed "quickening" is very far from satisfactory to our apprehension.

"We shall indeed cease to be surprised at this effect when we consider that from the uterus thus changing its situation, a very considerable pressure is suddenly removed from the iliac vessels, in consequence of which the blood rushes to the lower extremities, and a temporary exhaustion of the vessels of the brain, and a general loss of balance in the circulating system, are the results." 240.

Now, wherever there is pressure upon arteries and veins, both (and where can we make pressure without affecting both sets of vessels?) the veins, from their structure, experience most interruption of function, and consequently the parts behind the pressure are more than naturally turgid. Look at the lower extremities of a female when the advanced gravid uterus presses not only on the hypogastric but on the great iliac vessels. They are swelled, and often œdematous. The balance of the circulation then cannot be said to tend more to the head in the early than in the ulterior stages of utero-gestation, and consequently this explanation is not satisfactory on physiological grounds.

Why labour should come on at the expiration of nine calendar months, or about 40 weeks from the time of conception, we can give no better reason than that which was assigned in the time of Avicenna; namely, that—"it came on by the *command of God*"—a command that extends to every animal that brings forth, however various the periods of gestation.

Our authors now proceed to the consideration or solution of several physiological and forensic problems connected with the subject of pregnancy. And first, "*whether a woman can be delivered during a state of insensibility, and remain unconscious of the event.*" This must be answered in the affirmative. In certain comatose states of the brain—during the operation of strong narcotic substances—in the delirium of fever, such an event is not only possible but probable. Cases of the kind are on record ever since the days of Hippocrates.

2dly. *How far the term of utero-gestation can be shortened, compatible with the life of the offspring.* Capuron relates the case of Fortunio Liceti, who is said to have been born at the end of four months and a half, and lived to his 24th year. Our authors take no notice of the well authenticated case of a child born between the fourth and fifth month, and reared, at Paisley, in the year 1815, as recorded by Dr. Rodman in the 11th volume of the Edinburgh Medical and Surgical Journal. Such an authentic and excellent illustration happening in our own time and country should not have been overlooked. Physiologists of the present day consider that a fœtus born before the completion of the seventh month "has a very slender chance of surviving," although instances have occurred where the life has been preserved after a birth still more premature. Our authors ridicule, as a conceit, the idea entertained by Hippocrates, and even by many modern writers, that an infant could live at seven months, but rarely or never at eight. Whatever may be the degree of conceit in the business, we believe it is a fact that more children are born *and live* at seven than at eight months of utero-gestation.

Q. 3. *To what probable extent can the natural term of utero-gestation be prolonged?* The period of 40 weeks does not seem to be so arbitrarily established but that Nature may occasionally transgress her usual law, and over-run, or run short of, her reckoning.

"*Dr. Hamilton* remarks upon this occasion, that if the character of the woman be unexceptionable, a favourable report should be given for the mother, though the child should not be produced till nearly ten calendar months after the absence or sudden death of her husband." 246.

Upon this question, involving in its moral and legal relations the honour and happiness of families, the legitimacy of offspring, and the succession of property, both doctors of law and doctors of physic are divided. Mahon associates his name with Astruc, Mauriceau, Rhœderer, and Baudelocque, who reject the belief in retarded delivery as a thing impossible; while Fodere ranges with those who support the contrary opinion, among whom are Heister, Bartholin, Haller, Lietaud, Vicq. d'Azyr, and Capuron, to whom may be added Hippocrates, Aristotle, and Pliny. The Parliament of Paris, in the case of a widow, decided in favour of the legitimacy of an infant born in the 14th month after the death of her husband. The civil code of France has now placed a limit to our credulity respecting retarded births, and decrees 300 days or ten months to be the most distant period at which the legitimacy of a birth may be allowed. We agree with our authors that—"were we called upon to deliver an opinion upon this momentous question, we should certainly consider such a law as rather inclining on the side of mercy, than on that of stern justice."

Q. 4. *What is the value of those signs by which we seek to establish the fact of a recent delivery?*

"1. The face is pale, the eye sunken, and surrounded by a purplish or dark brown coloured ring; the pulse is full and undulating; the skin soft, supple, rather warmer than ordinary, and covered with a moisture which has a peculiar and somewhat acid odour.

"2. The breasts are swelled, are harder than usual, and painful; and, in some cases, lumpy to the touch, and emit, by pressure or suction, a lactiform fluid; the nipples are thicker, and the areola, by which they are surrounded, is widened in extent, and darkened in colour.

"3. The abdomen is flaccid, and its skin lies in folds, and is traversed in various directions with shining, reddish, and white lines, and light-coloured broken streaks, which especially extend from the groins and the pubes towards the navel.

"4. There is an extraordinary swelling and tumefaction of the external parts of generation; sometimes the anterior margin of the perineum is lacerated, or it is very lax, from the distention which it has undergone.

"5. The vagina is preternaturally distended; the orifice of the uterus is soft and open, and capable of admitting the point of the finger without difficulty, as if a late discharge had been made from it: the womb itself, not having properly collapsed, and resumed its natural shape and dimensions, may be felt through the parietes of the abdomen, voluminous, firm, and globular, contracting and expanding under the hand, on pressure.

"6. A discharge of serous fluid mingled with blood from the vagina, called the *Lochia*, continues from five to thirty-five days after delivery: it differs from the common menstrual flux in being paler, but more particularly in its peculiar and characteristic sour odour; at first this discharge is decidedly sanious, but in a few days it becomes of a much paler and of a brownish, or a dirty green hue, so as to acquire the common term of *green waters*." 252.

In the course of ten or twelve days, however, these signs become too obscure to afford unexceptionable evidence. It is proper also to consider the consecutive signs of parturition collectively, not individually, for as Chaussier has properly remarked—"no disease or affection besides parturition, can possibly produce the whole series of signs above described."

Q. 5. We are next to inquire if there be any, and what diseases, whose effects may be mistaken for traces of recent delivery?

Dropsical discharges from the uterus, uterine hæmorrhage, the expulsion of a mole, hydatid, or polypus—in short, the removal of any of those diseases which constitute what has been termed *false conception*, occasion effects which simulate the signs of parturition. Nor do we think our authors have pointed out any sure distinctions between the one case and the other, in the following passage:—

"The irruption of fluids from the womb, menorrhagia, and leucorrhœa, may mimic the *lochial* discharge, but they will not remain, nor will they present that characteristic odour by which the latter is so pre-eminently distinguished; so again, the relaxation of the soft parts may be the consequence of disease as well as of delivery, while the paleness of the visage is the usual concomitant of profuse evacuation; but then the distention of the vagina, and the state of the neck of the uterus, and the absence of all contusions, lacerations, and discolourations will obviate the possibility of deducing any erroneous conclusions from these phenomena; the wrinkles and marks upon the abdomen may certainly follow any considerable change in the reduction of its bulk, whether it be the result of parturition, ascitic discharges, or the absorption of fat; but we may easily disarm such signs of their treachery by a previous inquiry into the state of the woman's health, and into that of her robustness and general strength." 255.

Q. 6. *Can we determine, at a remote period, whether a woman has ever borne a child?*

In offering the following indications of a woman having borne a child, our authors properly remark that, *singly*, they can furnish but very slender evidence, and collectively they afford but strong presumption of the fact.

"1. The orifice of the womb has not its conic figure; its lips are unequal; and it is more open than in those who have never borne children.

"2. There is a roughness of the abdomen, the parietes of which are also more expanded and pensile.

"3. There are small white and shining lines running on the surface of the abdomen.

"4. The breasts are more flaccid, and pendulous, and the lines on their surface are white and splendid.

"5. The nipples are prominent, and the colour of their disks brown." 256.

Excepting the white lines on the abdomen, all the other marks being, as it were, *comparative*, are extremely equivocal. And even the abdominal lines might be produced by any cause that gave a temporary enlargement to the abdomen.

Q. 7. *To the question what are the earliest and latest periods of life at which women are capable of childbearing?*—we apprehend little positive can be said, unless we verge to great extremes. The commencement and cessation of menstruation are the natural boundaries; but these commence and decline at different periods in different individuals, and we imagine that it is by no means certain that a young female cannot conceive because she has not begun to menstruate—though the probabilities are against her, no doubt. Of the *extraordinary* and "well authenticated" cases on record we shall take no notice, as they are mere exceptions to a general rule.

Q. 8. *What is the possible number of children that can be produced at one birth?*

Twin cases occur on an average once in 90 labours—triplets about once in three thousand times—and quartettos are so rare that no calculation has been made on the subject. It is considered as a very wonderful fact, and a strong proof of Divine Providence, that the relative number of males and females born is nearly equal—21 males to 20 females. To us this fact does not appear a bit more wonderful than any of the other laws by which the universe, or even the human microcosm is governed. Is it more wonderful that the number of the sexes should be nearly equal, than that a black pudding should be converted into white chyme in the stomach of either sex?

Q. 9. *Is superfœtation possible—and at what period of gestation can it take place?* This question is more important in a legal than in a merely physiological point of view.

"A woman loses her husband suddenly, *tenant in tail male*, a month after marriage, and at a little more than eight months after his decease she is delivered of a perfect male child, and, at nine months, she declares that she is delivered of another infant, which is a male. The heir at law, who has entered, contests the fact of this latter birth; the question, therefore, to be determined is, whether such an event is compatible with the known laws of utero-gestation." 260.

We much doubt whether a court of law would seriously listen to the "well characterized example in the instance of Iphicles and Hercules, who were begat by Alcmena, the former by Jupiter, and the latter by Amphytrion."*

Hippocrates, Aristotle, Pliny, believed in superfœtation—and examples are recorded by the moderns corroborative of

* Vol. I. p. 261.

the possibility of such an event. That of Buffon's is the most in point.

"*Buffon* presents us with a still more striking example; a woman of Charles-town, in South Carolina, was delivered in 1714 of twins, which came into the world one directly after the other, but to the great surprise of the midwife, one was black and the other white; the woman herself, considering this proof of her infidelity too obvious to be denied, admitted the truth without hesitation,—that shortly after having enjoyed the embraces of her husband, a black servant entered her room, and by threats accomplished his purpose." 261.

Musa Brassavolus asserts that he saw an *epidemic* of this kind! "Nos vidimus super-fœtationem quandoque fuisse epidemicam affectionem." This is really satis superque! Haller was a strenuous believer in superfœtation. "Os uteri nunquam clausum est; ideoque potest super-fœtari non solum a die sexto ad trigessimum, aut primis duobus mensibus, sed omni omnino tempore." The case of superfœtation recorded by Dr. Maton in the 4th volume of the College Transactions, has been criticised by Dr. Granville, in the Philosophical Transactions, and defended in the present work by Dr. Paris. As the case was only orally related by Dr. Maton, we do not deem it necessary to enter into the dispute. The most probable cause of superfœtation is a septum in the uterus, which, in fact, renders it virtually a double organ.

Q. 10. *What are the causes of abortion?* Under this head reference is merely made to works on midwifery for information.

Q. 11. *Under what circumstances is it morally, legally, and medicinally proper to induce premature labour?*

The artificial induction of abortion is not described, as the work is designed for perusal beyond the pale of the profession. This is of little consequence, as the *modus* is well known to the surgeon. In cases of distorted pelvis, through which a full grown fœtus cannot pass, it becomes our duty to perform the operation for premature delivery. The following observation of Dr. Hull on this subject may not be unworthy of quotation.

"'The propriety of inducing premature labour,' says he, 'in any deformed woman, can rarely, if ever, be determined upon before the crotchet has been found indispensably necessary, and actually employed in a previous labour; indeed, unless the contraction of the tube or canal of the pelvis be very considerable and pretty accurately ascertained, it will scarcely be justifiable in any case to have recourse to this practice in all the subsequent pregnancies, until the woman has been delivered a second, or third time, by the crotchet; for it has happened in a very great number of instances, that a woman who has been delivered of her first child by the perforator and crotchet, has been afterwards delivered of one or more living children, at the full time; this observation is made not to discountenance the inducing of premature labour, but to prevent the abuse of it.' " 273.

Q. 12. *What circumstances will justify the Cæsarean operation, or the section of the symphysis pubis?*

Much learning, research, and irrelevancy, are unnecessarily employed in answering this and most other questions in the work before us. No pains, indeed, are spared to force in matter from historians, poets, and even dramatists. Here follows a specimen.

" It must be admitted, that a child taken from the womb of its mother by the cæsarean section, cannot, in philological strictness, be said to have been *born*. The ingenious purpose to which *Shakspeare* has applied this quibble has no doubt suggested itself to the reader.

" *App.* Macbeth! Macbeth! Macbeth!
* * * * * *
Be bloody, bold, and resolute; laugh to scorn
The power of man; for *none of woman born*
Shall harm Macbeth. *Act* iv. *sc.* 1."

" *Macd.* * * Despair thy charm;
And let the angel, whom thou still has served,
Tell thee, *Macduff was from his mother's womb*
*Untimely ripp'd.* *Act* v. sc. viii." 281.

The whole marrow of this section might be contained in a nutshell. The operation ought not to be performed " where by embryulcia the child can be extricated." And again, " In the event of a woman, near the full time of pregnancy, dying undelivered, the Cæsarian operation ought always to be performed with as little loss of time as possible." This is the sum and substance of many pages.

### EXTRA-UTERINE PREGNANCY.

This subject is introduced without even a hint that it has any connexion with medical jurisprudence. It is like the spleen, a mere locum tenens to fill up two or three pages. Calculus or gall-stones would have done just as well.

### HERMAPHRODITES.

Although there can be no doubt that some of the lower order of animals are, in the strictest sense of the term, hermaphrodites, " it is now universally admitted that, in the human species, no such phenomenon ever existed." " Nor is there upon record a single case which can be considered authentic." It is, of course, in cases of this kind, where preternatural structure gives the appearance of a double sex, that medical men are called upon to decide. Sir Everard Home considers that all the monstrous productions, hitherto described as hermaphrodites, may be reduced to one of the four following classes:—

"1. Malformations of the male. 2. Malformations of the female. 3. Males with such a deficiency in their organs, that they have not the character and general properties of the male, and may be called NEUTERS. 4. Where there exists a real mixture of the organs of both sexes, although not sufficiently complete to constitute double organs." 285.

### IDIOTS AND LUNATICS.

The first eighteen pages of this section are occupied with *law*, scarcely any part of which can our poor intellects comprehend. For our own parts, we are determined, whenever it shall please God to afflict us with *insanity*, to put the business into a lawyer's hands—but, while we are *compos mentis*, we shall have nothing to do with law. The physician dabbling in law is something like the lawyer dabbling in physic—they will both botch the business. "Ne sutor ultra crepidam," say we.

In commencing the medical and physiological illustrations of insanity indeed, it is acknowledged that, "the duties of the jurist and physiologist in the investigation of mental derangement are distinct in their nature, if not different in their object." Hence it is that the abstract terms used to denote the form or degree of the malady have received from the two professions a somewhat different latitude of acceptation.

"For legal purposes the adoption of the term "*Non Compos Mentis*," from the amplitude of its construction, gets rid of those nicer distinctions and difficulties which the pathologist is bound to encounter and investigate; the lawyer only inquires whether such a state of mind exists, as actually disqualifies the person in question from conducting himself with propriety, or managing his affairs; but the medical evidence is bound not only to give his opinion upon the case, but to state the reasons which may have influenced his decision; and hence the necessity of his becoming practically acquainted with those physiological distinctions to which we have alluded." 307.

There are two conditions of the human mind, either of which very justly deprives the subject of the control of his person and property, and takes from him all criminal responsibility, viz. Idiotcy, or total deficiency of intellectual power; and madness, or a morbid perversion of it.

"Between these two states we shall not have much difficulty in discriminating; the idiot cannot reason at all; the madman reasons falsely; the idiot acts from animal appetency, he has no will; the madman wills, but his reason being disturbed, his actions are not compatible with the usual relations of society." 308.

The medico-legal questions which our authors propose to agitate in this work are as follow:—

"Q. 1. Whether the person be actually insane? and what are the proofs of his derangement?

Q. 2. Whether the symptoms are of such a nature as to suffer the individual, with propriety, to retain his liberty, and enjoy his property.

Q. 3. Whether there has been any lucid interval, and of what duration?

Q. 4. Whether there is a probable chance of recovery; and in case of convalescence, whether the cure is likely to be permanent?" 317.

In respect to the *first* query, it has been justly remarked, that to constitute insanity it is not necessary to exhibit the ferocity of a wild beast, nor to perform the antics of a buffoon. The most ordinary observer can tell when a man is furiously mad; but in many cases—"such thin partitions do the bounds divide," that all the skill and discernment of a medical practitioner is required to establish the existence of insanity. It is difficult to lay down any rule for the investigation of a case of real or supposed insanity. The practitioner must make himself acquainted with the pathology and history of the disease in the proper treatises, *ex professo ;* for little or nothing of course, is to be learnt on the subject from a book like that before us.

"'It is nearly impossible,' says *Dr. Haslam*, 'to give any specific directions for conducting such an examination as shall inevitably disclose the delusions existing in the mind of a crafty lunatic; but in my own opinion it is always to be accomplished, provided sufficient time be allowed, and the examiner be not interrupted. It is not to be effected by directly selecting the subjects of his delusion, for he will immediately perceive the drift of such enquiries, and endeavour to evade, or pretend to disown them; the purpose is more effectually answered by leading him to the origin of his distemper, and tracing down the consecutive series of his actions and association of ideas; *in going over the road where he has stumbled, he will infallibly trip again.*'" 319.

Nor is it always easy to discriminate between eccentricity and insanity. Do we not, as Dr. Male observes, see a wretch disinherit his own children, who have committed no fault, and bestow his wealth upon a stranger? Another who prefers poverty and rags, and communion with vagabonds, to the social intercourse and proffered kindness of his friends and relations? yet, who shall pronounce them insane? That they are so there can be no doubt; and their disease is perhaps of the most unfortunate character: for all their other actions being consistent with sound reason, it is difficult to convince a jury of their insanity, and to divest them of the power of heaping ruin upon their families, and disgrace upon themselves.

"The bodily marks which distinguish the insane are, a peculiar cast of countenance, familiar to those versed in the malady; a quick, oftentimes protruded and glistening eye; the body is generally costive; in some cases the insane person is enabled to sustain cold with impunity, and he is insensible to the agency of ordinary stimuli; and the stomach and bowels, from deficiency of irritability, require large doses of medicine to move them; among the physical phenomena of insanity, *M. Esquirol* observes that few are more constant or remarkable than want of sleep, and that peculiarly disagreeable odour from the body, as well as the excretions of the patients, which impregnates the clothes and bedding. They are devoured with a burning internal heat; and generally have a voracious appetite, and are afflicted with pain in some organ or part, especially the head, the chest, or the abdomen, which the unhappy sufferers are ready to attribute to the malevolence of their enemies." 320.

The *second* query cannot be answered by our authors, "as each case must rest on its own merits."

On the *third* query respecting the existence of lucid intervals, it is remarked, with truth, that the subject is beset with difficulties; for, in many cases, the patient can dissemble so well, for a limited period, as to appear perfectly rational, although the hallucination is deeply fixed in his mind. The following case from Haslam will elucidate this observation.

"'A lunatic having received, or fancied he had received, an injury from his keeper, at the lunatic asylum at Manchester, threatened to be revenged, for which he was punished by confinement; he was afterwards a patient in Bethlem Hospital, and gave *Dr. Haslam* an account of the transaction, of which the following is an abbreviation. 'Not liking this situation, I was induced to play the hypocrite; I pretended extreme sorrow for having threatened him, and, by an affectation of repentance, induced him to release me; for several days I paid him great attention, and lent him every assistance; he seemed much pleased with the flattery, and became very friendly in his behaviour towards me: going one day into the kitchen, where his wife was busied, I saw a knife; this was too great a temptation to be resisted; I concealed it, and carried it about with me; for some time afterwards the same friendly intercourse was maintained between us, but as he was one day unlocking his garden-door, I seized the opportunity, and plunged this knife, up to the hilt, in his back.'" 323.

The *fourth* question on the probable chance of recovery must be resolved by taking into consideration a variety of circumstances respecting the modification of the malady, the violence of the symptoms, the duration and frequency of attacks, &c. When mania has acquired a systematic character, it is very difficult to remove it; so that after it has continued upwards of a year, patients at asylums, as Bethlem and St. Luke's, are pronounced incurable, and treated accordingly. After the various articles on insanity which have appeared in this Journal, it would be needless to advert to the scanty information on its pathology, which meets the eye in the work before us.

NUISANCES.

Although there are many kinds of nuisances, our authors properly confine themselves to those which can be made the subject of medical or chemical investigation. These are such as are directly or indirectly detrimental to health, whether general or individual—destructive to comfort—injurious to property—obstructions to the free course of light, air, water, &c.

"The question, how far the salubrity of the atmosphere may be affected by the effluvia of particular manufactories, is one that the medical practitioner is often called upon to decide; and upon such an occasion let him beware that his judgment be not swayed by the fastidiousness of the surrounding inhabitants, nor warped by the clamours of invidious rivals or interested opponents; as a man of science and integrity he is called upon to decide between two parties equally valuable to the state,—between the health and comfort of the citizen, and the prosperity of the manufacturer." 330.

The manufactories which have been considered exceptionable are arranged by our authors under four heads.—1st. Those extricating gaseous effluvia, the products of *putrefaction or fermentation*, as flax-steeping—cat-gut manufacture—slaughter-houses—tanneries—skinners, fell-mongers, curriers, &c. 2d. Those where fire is the agent of extrication, as brewing, distilling acids, incineration of animal substances, glue-making, horn-roasting, soap-boiling, gas-making, &c. 3d. Those which yield waste liquids that taint the neighbouring streams, as gas works, dying-houses, &c. 4th. Noisy trades, as copper-smiths, anchor-makers, gold-beaters, tin-men, &c. &c. We shall here introduce the observations of our authors entire, on this curious and interesting subject. They are in the best stile of our authors, and they combine entertainment with instruction.

"On the manufactures and occupations above alluded to, we have to make the following observations.—

"(1) As the vegetable matter undergoes the putrefactive process in stagnant pools, the effluvia which arise are necessarily highly pernicious; while the waters become so poisonous as to destroy the fish contained in them, as well as to prove injurious to cattle that drink of them. In Italy the process of steeping flax or hemp is only permitted at the distance of some leagues from a town. ZIMMERMAN tells us that the effluvia from this source have been known to occasion a malignant fever, which proved fatal to the family in which it first began, and afterwards spread its contagion through a whole country. LANCISI observes, that dangerous fevers are often prevalent at Constantinople, which owe their source to the hemp brought from Cairo, and which is put wet into the public granaries, and suffered to ferment during the summer. At Helmstedt there is annually in the autumn, when the flax is steeped in the Aller, an epidemic dysentery that prevails for several weeks.

"(2) The manufacture of starch can scarcely be considered, in itself, a nuisance, for although it be necessary to produce the acetous fermentation, in order to remove from the fecula any colouring matter, yet if sufficient attention be paid to the operation, and the water be properly let off from the settling-vessels, no inconvenience can arise. A nuisance, however, of considerable magnitude may incidentally attend these manufactories, from the number of swine which are constantly kept by the starch maker, and the profit of which forms a part of his speculation, and which is so considerable that he can generally afford to sell the starch at prime cost, relying wholly upon the former trade for his profits.

"(3) The process of tanning involves several operations of a very nauseous description; the hides, for example, undergo incipient putrefaction in order to loosen the epidermis, and to render the hair and other extraneous matter easy of separation from the true skin.

"(4) The peregrinations and vicissitudes of fate to which the horse is doomed during life has repeatedly furnished subjects of reflection; but few are aware to how many economical purposes his carcase is converted after death, and to how many noisome processes it gives rise. The dealers in dead horses, or *nackers*, as they are termed, begin their mercantile anatomy by taking off the shoes and disposing of them to the farrier; the skin is next stripped off, and sold to the tanner; the carcase is then cut into pieces, and boiled in large cauldrons of water, in order to extract the fatty matter, which, being skimmed off from the surface of the liquor, is '*rendered down*' and packed in cases for the soap-boiler, or the manufacturer of cart-grease. Whatever remains after this operation supplies the venders of dog's and cat's meat with a dainty article of sale; at length the views of the greedy trader are directed to the bones of this noble animal; a number of persons find employment in chopping them into small fragments, from which the marrow is then extracted by boiling for several hours, and added to the fat already obtained from the carcase; the dry remains are employed in the production of hartshorn by distillation; and after this process is finished, they are removed from the still, and calcined to whiteness, in order to be mixed with clay for the manufacture of porcelaine; or they are consumed for the formation of ivory-black.

"(5) The intolerable nuisance of a public brewery arises from the volumes of carbonaceous matter with which it overwhelms the neighbourhood. We shall therefore take this occasion to offer the remarks which we are prepared to make respecting the effects of smoke on the inhabitants of the metropolis, and on the methods which have been suggested for the mitigation of the evil. And upon this subject we entirely agree with an intelligent reviewer, that, after all, it is not a few chimneys attached to steam engines that infect the air of London with smoke; every house is busy in the work of contamination, although less observed, because administered by separate vents, and in divided doses.

"In the year 1661, a work was published by the celebrated John Evelyn on the subject of this grievance, entitled, 'Fumifugium; *or the Inconveniences of the Air and Smoake of London dissipated; together with some remedies humbly proposed to his sacred Majestie, and to the Parliament now assembled.*' The above 'short discourse' has become exceedingly scarce, but the reader will find an interesting account of its contents in the Journal of Science and the Arts. It is certainly a curious coincidence that the attention of John Evelyn should have been first excited on this subject by 'a presumptuous smoake issuing from one or two tunnels near Northumberland-house, and not far from Scotland yard,'—the very seat of the plots of our modern fumifugists! After adverting to the situation of the metropolis 'built upon a sweet and most agreeable eminency of ground at the north side of a goodly and well conditioned river, toward which it has an aspect by a gentle and easie declivity,' he proceeds to animadvert upon that 'hellish and dismall cloud of sea coale, which is not only perpetually imminent over her head, but so universally mixed with the otherwise wholesome and excellent air, that her inhabitants breathe nothing but an impure thick mist, accompanied with a fuliginous and filthy vapour, which renders them obnoxious to a thousand inconveniences, corrupting their lungs, and disordering the entire habit of their bodies.' It appears that in Evelyn's time, brewers, dyers, lime-burners, and salt and soap-boilers, were the principal nuisances; and 'since then,' says the editor of the new edition of the Fumifugium in 1772, 'we have a great increase of glass-houses, founderies, and sugar-bakers, to add to the black catalogue, at the head of which must be placed the fire engines of the water-works at London bridge and York-buildings, which leave the astonished spectator at a loss to determine whether they do not tend to poison and destroy more of the inhabitants by their smoke and stench than they supply with water;' to which sooty list, says the reviewer, in the Journal of Science and the Arts, above cited, 'what astonishing additions have been made, within the last thirty years, in and about London? How many new water-companies, and smoke-producing manufactories have been added to the catalogue? A newspaper cannot now be printed, nor a pound of meat minced for sausages without a steam-engine; to the same smoky servant the druggist resorts to grind his rhubarb and to sift his magnesia,* and upon all possible occasions the services of the other elements is superseded by that of fire.' With respect to the deleterious effects of smoke upon the health of the inhabitants of our mighty city, much difference of opinion has existed; amongst Foreigners the air of London has the reputation of being extremely unhealthy, on account of the exhalation which arises from the use of coal; it excites in strangers, says *Zimmerman*, a considerable heat in the stomach, and sometimes a spitting of blood, and even nervous fevers which terminate in palsy. (*Experience in Physic, vol.* 2, *p.* 137). It is hardly necessary for us to make any observation upon a prejudice so absurd and unfounded; *Evelyn* also seems, in our opinion, to attribute more evils to the smoke than can be well substantiated; 'I report myself,' says he, to all those who have been compelled to breathe the air of other countries for some years, if they do not now perceive a manifest alteration in their appetite, and clearness of their spirits, especially such as have lived long in France and the city of Paris.' Although we are not disposed to consider the smoky atmosphere of London as so destructive to health as some have imagined, we are not prepared to state that it is entirely harmless. Children are certainly less healthy in this city than in the country; and the superior rapidity with which iron becomes oxidized, indicates the existence of atmospheric impurities. The phenomena of vegetation also offers another demonstration of the same fact; *Evelyn* has the following curious remarks upon this circumstance; 'That the smoake destroys our vegetation is shewn by that which was by many observed in the year 1664, when Newcastle was besieged, and blocked up in our late wars, so as through the great dearth and scarcity of coals, these fumous works were either left off, or diminished, divers gardens and orchards planted in the very heart of London, (as in particular, my Lord Marquis of Hertford in the Strand; my Lord Bridgewater's, and some others about Barbican) were observed to bear such plentiful and infinite quantities of fruits, as they never produced the like before, or since, to their great astonishment; but it was by the owners rightly imputed to the penury of coales, and the little smoake which they took notice to infest them that yeare.'

"Although some difference of opinion may exist, as to the extent of the evil, in a medical point of view, we must all concur in agreeing upon the necessity of some plan by which it may be diminished; we

---

* "By a visit to Apothecaries' Hall, or to any of the great manufacturing chemists, the stranger will be astonished at the number and utility of the applications of steam to the process of Pharmacy.

"*M. Dupin*, when speaking of the immense mechanical force set in action by the steam-engines of England, gives the following illustration of its amount:—The great pyramid of Egypt required for its erection the labour of above 100,000 men for twenty years; the action of the steam-engines in England, which are, at most, all managed by 36,000 men, would be sufficient to produce the same quantity of work, in 18 hours!!!

shall, therefore, proceed to offer some remarks upon the proposals which have been made, at different times, for obtaining so desirable an object.

"*Mr. Evelyn's* plan consisted in the removal of all smoking manufactories from London, 'five or six miles down the river Thames, or at least, so far as to stand behind that promontory jutting out and securing Greenwich from the pestilential air of Plumstead marshes.' He then proposes gardens and plantations in and about the metropolis, and enumerates a variety of fragrant plants, suited to our climate, and calculated to sweeten and improve the air.*

"In the year 1682, Mr. *Justell* communicated to the Royal Society, '*An account of an Engine that consumes smoke, shewn lately at St. Germains Fair in Paris.*' *Dr. Leutmann*, of Wirtemburgh, described in his 'Vulcanus Famulans,' a stove which draws downwards, so that the contrivances of the *Marquis de Charbannes*, and others who have burnt their smoke by a downward draught of air were not original. *Dr. Franklin* in 1785 (*Memoirs of the Life and Writings of Benjamin Franklin, vol.* iv. *p.* 408) suggested a mode of burning smoke; but to the illustrious *Mr. Watt*, we are more particularly indebted for the first important hints upon this subject; his patent may be seen in the *fourth volume of the Repertory of Arts* for 1796, *p.* 226; and the great engines at the Soho manufactory have all along been worked without smoke; it is therefore not a little extraordinary, as a late reviewer has justly observed, that in the Report from the Committee of the House of Commons 'to enquire how far it may be practicable to compel persons using steam engines and furnaces in their different works to erect them in a manner less prejudicial to public health and public comfort,' and upon which report the bill of last session is founded, no notice is taken of *Mr. Watt's* suggestions and inquiries. In the Parliamentary Report, to which we have just alluded, there are two inventions for the destruction of smoke, which appear to have principally occupied the attention of the Committee, and which profess to accomplish the object, with a very considerable saving of fuel, viz:

"*Mr. Brunton's Fire Regulator.* In this patent a newly constructed fire-place is applied to the engine boiler, containing a circular grate, which is made to revolve slowly upon its axis; the fire upon this grate is fed in front by a kind of hopper continually delivering small coal, which, from the rotatory motion of the grate itself, becomes equally spread upon its surface, so as to maintain a thin fire, and a sharp draught; the coal is thus rapidly decomposed and burned; the smoke at first produced having to pass across the grate, and over the red hot, and already coaked fuel.

"*Patent of Messrs. Parkes of Warwick.*—The principal agent in this improvement is a current of air, admitted just beyond the end of the fire place, by means of an aperture which may be increased, or

---

* "It is supposed that the lime-trees in St. James's Park owe their existence to the suggestion of Evelyn."

closed at pleasure, and which the patentee term an '*air valve.*' A small fire is first made to burn brightly at the back of the grate; coals are then filled in towards the front, in which direction the fire gradually spreads; their smoke necessarily passes over the clear fire, where it becomes sufficiently heated to constitute flame, as soon as it meets with the current of air entering at the valve; and a striking experiment with this apparatus consists in alternately shutting and opening the air-valve, which is accompanied by the alternate appearance and disappearance of the smoke.

"Instead, however, of insisting upon any form of fire-place, greater benefit would arise from an enactment respecting the height of chimnies; our intelligent reviewer, of whose remarks we have so frequently availed ourselves, observes, that by conveying black smoke, and other pernicious fumes into a capacious and very lofty chimney, much of the noxious matters that otherwise escape into the atmosphere are decomposed and precipitated or condensed within; of the truth of which, the chimney of the grand-junction engine, at Paddington, and that of the West Middlesex water-works, at Hammersmith, offer striking illustrations; when these machines are at work, the former produces little smoke, while the latter inundates the neighbouring gardens with perpetual showers of solid soot; and yet the only difference is in the relative altitude of the two chimneys; the boilers being, in all respects, set and constructed alike. A chimney from 150 to 200 feet would in most cases prove effectual, and the expense might be considerably lessened by making one shaft receive all the tributary fumes of many flues. But to return to the nuisance of breweries, from which we have made so long a digression; it is probable, that the smoke from these chimneys could not be remedied either by Brunton's or Parkes's patent, but the increasing the altitude of the chimney would seem to promise a mode of relief; we are also to look to the employment of steam as a substitute for fires; high pressure steam has been very extensively employed for this purpose in Whitbread's brewery, and the smoke has in consequence sustained a very perceptible diminution.

(6) "Sulphuric acid makers are continually indicted; and it would appear that by a scientific improvement in the process, the escape of the sulphurous acid, which constitutes the grievance, might to a great degree be obviated. How does it happen that, notwithstanding the cost of the materials necessary for the production of sulphuric acid, is in France at least double what it is in England, the French can afford to sell the article 25 per cent. cheaper than the English? the answer is obvious,—the great part of the materials are sent off into the air, in the form of *sulphurous acid*, and *nitrous gas*, to the annoyance of the neighbouring animals and vegetables, and the ruin, too often, of the proprietor. See *Journal of Science and the Arts.* In a report drawn up in the year 1806 by Guyton Morveau, and Chaptal, upon the subject of injurious manufactories, by command of the minister of the interior, it is declared that the distillation of acids can only prove dangerous from want of due precaution.

"(7) The manufacture of Prussian blue is necessarily attended with highly offensive vapours. The first part of the process consists in mixing hoofs and tups' horns with Russian or American potass, in large iron stills, to which heat is gradually applied, until the vessel become red hot; the animal matter and alkali being thus fused into a mass is laded out into iron pans, where it concretes into solid blocks, technically called *metals.*

"(8) The operation of unfolding the cows' horns by the application of heat is attended with a terrible stench; the trade in lanthorn leaves was formerly very considerable with Russia; but it was nearly annihilated by an edict of Catherine; the less flexible parts are made into combs; and the tips of the horns are sent to Birmingham for the manufacture of buttons.

"(9) Owing to the viscid nature of the materials, it is impossible to make varnish without burning the animal matter, which occasions a stench of the most insufferable kind; and is so suffocating, that very lately two workmen lost their lives in a manufactory of this article in Gray's-inn-lane.

"(10) The animal matters employed in this process give rise to a stench which has repeatedly formed the ground of indictment. The most nauseous part of the trade, however, consists in concentrating the waste lees, for the purpose of obtaining by fusion in a reverberatory furnace, an article which is called BLACK ASH, and which contains, amongst other salts, the *sulphuret of soda.*

"(11) 'Renderers of tallow' are persons who convert the butcher's fat, &c. into tallow.

"(12) The process of smelting different ores is the most injurious of all the operations of art, although to the senses it may be less nauseous than those in which animal matter undergoes decomposition by heat, or putrefaction. These evils, however, by the ingenious application of various mechanical and chemical expedients, have in many instances been very materially diminished, and in others entirely obviated; this is strikingly illustrated in several large works for smelting lead ores; and the proprietors of the Hafod copper works, at Swansea, are at present engaged in an experimental inquiry into various plans which have been proposed for diminishing, or preventing the ill effects which arise from the metallic fumes. Acquainted as we are with the liberality and science of these gentlemen, we have little doubt of the result; and we mention the circumstance in this place in order to recommend similar efforts on the part of persons engaged in other works; and at the same time for the purpose of preparing the reader for some observations which we shall take occasion to offer, on the subject of the law of nuisance, in relation to its operation in stopping works of such national importance. It would be premature to enter into any detailed account of the chemical means which promise a successful resource on this occasion; we shall only observe that the great mischief seems to arise from the quantity of asenic, so universally present in the ores of copper; and there is reason to hope, from the experiments already made by Mr. J. H. *Vivian,* that *Lime* may be usefully employed in preventing its volatilization. The author of the present note has had ample opportunities of investigating the effects of arsenical fumes, which arise from the burning-houses in Cornwall, and from the great copper works carried on at Hayle in that county, and they appear to be especially pernicious to graminivorous quadrupeds; horses and cows lose their hoofs; and the latter animals are not unfrequently seen, in the vicinity of the works, crawling along on their knees; they are also subject to a cancerous affection in their tails; and milch cows lose their milk. The herbage also suffers materially from the poisonous smoke, especially in wet seasons; corn is blighted in the ear, and never perfects its seed, unless care be taken to select at that period such ore as will yield but little sublimate. Cabbages do not appear to suffer in the least; nor are potatoes materially injured; and it is not the least curious circumstance in the history of these works, that the apple-trees in their vicinity grow and bear fruit without sustaining any of those ill effects which we should have anticipated, but, on the contrary, the arsenical fumes appear to destroy all the insects which usually infest such trees, and their trunks exhibit a cleanness which would delight the horticulturist. The men employed in these works are occasionally affected with a cancerous disease in the scrotum, similar to that which infests chimney-sweepers; it is however probable that this arises from the immediate application of the excoriating material made by the hand in the act of rubbing the part. A similar affection was a short time since observed in a manufactory, in which the workmen were engaged in making an arsenical solution for a green dye, used in calico printing.

"(13) Gas Works. We have lately learnt, that a method has been adopted to get rid of the nuisance which has arisen from the residual liquor from these works, by evaporating it in pans, placed in the ash-pit of the furnace, and by which the iron bars of the fireplace are at the same time kept cool, and are therefore much longer preserved. The contrivance may be seen at the gas works in Worship Street." 331 to 339.

Here we close our first analytical article. Our readers will easily have formed, in the course of our review, a tolerably correct estimate of the nature and merits of the work. Our own opinion, which, however, is of no more importance than that of any other individual who reads or examines the publication, may be conveyed in two words. It is executed with considerable ability as far as the *medical* literature and science are concerned; but the whole is so diluted, or we might say adulterated, with useless or irrelevant matter, as to render the publication far less creditable to the authors and more expensive to the public than it would have been, were it composed in a terse didactic style, and on a plan that rigidly excluded meretricious ornament and tiresome superfluity.

