# Errata

**Published:** 1823-12-01

**Authors:** 


### ERRATA.

396 *line* 18 *for* Etymoligies *read* Etymologies.

